# Can the health effects of widely-held societal norms be evaluated? An analysis of the United Nations convention on the elimination of all forms of discrimination against women (UN-CEDAW)

**DOI:** 10.1186/s12889-019-6607-6

**Published:** 2019-03-08

**Authors:** Christopher A. Tait, Ifrah Abdillahi, Wendy Wong, Heather Smith-Cannoy, Arjumand Siddiqi

**Affiliations:** 1 Dalla Lana School of Public Health, University of Toronto, 155 College Street, Toronto, ON M5T 3M7 Canada; 2 Department of Political Science, University of Toronto, 100 St George Street, Toronto, ON M5S 3G3 Canada; 3 Department of International Affairs, Lewis & Clark College, 0615 S.W. Palatine Hill Road, Portland, OR 97219 USA; 4 Department of Health Behavior, Gillings School of Global Public Health, University of North Carolina – Chapel Hill, 135 Dauer Drive, Chapel Hill, NC 27599 USA

**Keywords:** women’s health, Human rights, CEDAW, Global health, Norms, United Nations

## Abstract

**Background:**

Female life expectancy and mortality rates have been improving over the course of many decades. Many global changes offer potential explanations. In this paper, we examined whether the United Nations Convention on the Elimination of All Forms of Discrimination Against Women (CEDAW) has, in part, been responsible for the observed improvements in these key population metrics of women’s health.

**Methods:**

Data were obtained from the United Nations Treaty Series Database, the World Bank World Development Indicators database and, the Polity IV database. Because CEDAW is nearly universally ratified, it was not feasible to compare ratifying countries to non-ratifying countries. We therefore applied interrupted times series analyses, which creates a comparator (counterfactual) scenario by using the trend in the health outcome before the policy exposure to mathematically determine what the trend in the health outcome would have been after the policy exposure, had the policy exposure not occurred. Analyses were stratified by country-level income and democratization.

**Results:**

Among low-income countries, CEDAW improved outcomes in democratic, but not non-democratic countries. In middle-income countries, CEDAW largely had no effect and, among high-income countries, had largely positive effects.

**Conclusions:**

While population indicators of women’s health have improved since CEDAW ratification, the impact of CEDAW ratification itself on these improvements varies across countries with differing levels of income and democratization.

**Electronic supplementary material:**

The online version of this article (10.1186/s12889-019-6607-6) contains supplementary material, which is available to authorized users.

## Background

During the mid 2000s, the World Health Organization Commission on Social Determinants of Health codified the notion that societal conditions – political, economic, social – are the ‘causes of the causes’ of population health, because they lie at the root of how (and to whom) health resources are distributed in society [1, 2]. Research before and since has examined impacts on health of a range of societal conditions, including social policies and recessions and their associated austerity responses [3–5]. Overwhelmingly, the main factor that has enabled the quantification of the effects of these societal conditions is the fact that they vary across countries or across groups in society. This variability has allowed researchers to use the classical epidemiological approach of comparing ‘exposed’ and comparator ‘unexposed’ individuals or societies [6]. However, some societal conditions hardly vary at all, posing a challenge for measuring their effects. And yet, these conditions may indeed be important determinants of population health. In this paper, we examine one such societal condition, the United Nations Convention on the Elimination of All Forms of Discrimination Against Women (CEDAW), and estimate its association with women’s mortality rates and life expectancy, two key population indicators of women’s health [7].

The CEDAW convention was adopted by the United Nations in 1979, and is “…often described as an international bill of rights for women…” [7]. The convention is intended to protect women from sex-based discrimination, and provide them with “...human rights and fundamental freedoms in the political, economic, social, cultural, civil or any other field [7].” Signatories to the convention commit to upholding the convention’s 30 articles. By accepting the Convention, states commit themselves to undertake a series of measures to end discrimination against women in all forms, include incorporating the principle of equality between men and women in their legal system, abolishing all discriminatory laws and adopt appropriate ones prohibiting discrimination against women, establishing tribunals and other public institutions to ensure the effective protection of women against discrimination; and ensuring the elimination of all acts of discrimination against women by persons, organizations or enterprises [7].

In theoretical terms, because CEDAW was intended to improve the status of women, it might therefore be expected to improve women’s health. The general public health literature posits that such societal conditions are, effectively, the deepest of root causes of health. The idea is that they determine a broad range of economic and social circumstances, which then influence and trigger a variety of behavioral and biological mechanisms, which are ultimately expressed as health outcomes [2]. For example, income assistance policies are a ‘societal condition’ that are a significant determinant of the material circumstances of the poorest in society. In turn, material circumstances influence access to nutritional foods, experiences of stress, and so on, and these manifest in health outcomes as varied as hypertension and depression.

Further conceptual work by Moss [8] has specifically explicated how and why a societal condition such as CEDAW should be expected to improve the health of women. As she suggests, by addressing discrimination against women, gender equity initiatives improve the material conditions of women and, through improvement of material conditions, as well as independent of these improvements, they increase the status of women in society. For example, CEDAW can lay the foundation for policies which access to education for girls and women. It can promote more equitable intrahousehold allocation of resources between husbands and wives and between girl- and boy- children. It can also change norms in society regarding the status of women. Their increased material well-being raises their status, but, independent of these changes, the sheer fact of a state’s commitment to addressing gender discrimination also sends a broad signal regarding the importance of fair treatment for women. In turn, these material and social gains can improve women’s access to health resources, such as nutrition and income. It can reduce the psychological and physiological stress that are triggered by discrimination and lower social status, and result in improved health outcomes for women [9, 10].

Indeed, gains to female life expectancy and declines in female mortality rates have been observed since treaty ratification [1, 11–15]. According to World Bank data, these indicators of women’s health have nearly uniformly been rising across countries, with exceptions due to specific shorter-term circumstances or particular subgroups (e.g., AIDS epidemic in sub-Saharan Africa, black maternal mortality in the United States) [16].

However, since the time that CEDAW first was introduced, other important societal conditions have also been simultaneously changing, such as growing economic development and declines in poverty rates, that may also account for improvements in women’s health, and can confound our understandings of the impact of CEDAW, independent of these other secular changes.

Moreover, most common statistical methods that are routinely used to isolate the effect of one societal condition from other co-occurring factors cannot easily be applied to isolate CEDAW from other societal conditions. This is because these methods rely on variation across societies in societal conditions, and use this variation to compare societies with a particular societal condition to those without it [6]. For example, the effects of post-recession austerity measures have been studied by comparing societies that implemented austerity measures to those which did not [17, 18].

By contrast, CEDAW, by and large, does not vary across societies. It has been widely ratified by countries around the world. In the political science literature, it has become what is known as an international ‘norm’ [19]. Few remaining countries have yet to ratify CEDAW. These include Iran, Somalia, Sudan, and the United States. This presents a happy nuisance – a sign of progress on women’s rights, but a methodological challenge to isolate the impact of CEDAW on women’s health.

In this paper, we present an analysis of the association between CEDAW and female life expectancy and mortality rates using a set of statistical methods that are not often applied in the population health literature, but which provide an opportunity to estimate the health impact of societal conditions, such as CEDAW, which have become societal norms. Our study thus can also be viewed as a case study for evaluating ‘norms’ and other societal conditions that do not demonstrate a high degree of variation within or between societies. We hypothesize that, across countries, CEDAW will be associated with improvements in women’s health, as evidenced by a steeper rise in female life expectancy and a steeper decline in female mortality rates after, compared to to before, its ratification.

## Methods

### Data sources

The following datasets were merged in order to assemble the required variables on CEDAW ratification, health outcomes, and relevant confounders and effect-modifiers: (a) the United Nations Treaty Series Database, which contains information on treaty ratification, (b) the World Bank World Development Indicators database, which contains metrics of population health and general economic indicators and, (c) the Polity IV database, which contains information on political democratization. Of the 193 countries for which we had data on ratification status, 187 (99.5%) had ratified CEDAW. Non-ratifying countries were excluded. Our final analytic sample consisted of 187 countries over the period 1980–2015, during which we had complete information for all countries.

### Measures

Our primary outcome measures were country-level female life expectancy, which was measured as projected average longevity at birth, and female mortality rate, which was measured as deaths among women per 100,000 women in the population.

We also sought to include measures of other country-level characteristics, which could either confound or modify the effects of CEDAW. Because of the limited sample size, we were only able to include key, summarizing characteristics, rather than a more exhaustive set of specific indicators. The characteristics we included were country-level income and level of political development (whether a country is a democracy or not). The notion was that higher income and more democratized countries, with better strength of institutions and infrastructure, may have greater availability and accessibility of education, health care, and other important health resources. On the one hand, these are factors that have a positive impact on women’s health, independent of what is happening to discrimination against women, and thus the observed effect of CEDAW may be in part an effect of these other institutions and infrastructure (a confounding or selection bias effect). On the other hand, it could be that an environment in which there is greater strength of institutions and greater democracy better facilitates a state effort to reduce discrimination against women (an modification of the effect of CEDAW).

We measured economic development using per capita gross national income (GNI) adjusted for purchasing power parity using the World Bank Atlas method. The Atlas conversion factor for any year is the average of a country’s exchange rate for that year and its exchange rates for the two preceding years, adjusted for the difference between the rate of inflation in the country and international inflation. The overall objective of this method is to reduce any changes in the exchange rate that are attributable to inflation. Based on GNI, economies were divided into the World Bank’s income groupings: low, lower-middle, upper-middle, and high. Due to sample-size restrictions, we collapsed the lower-middle and upper-middle income countries into one category of middle-income countries. For descriptive purposes, we also classified countries according to the World Health Organization’s 6 regions including: Africa, Americas, Eastern Mediterranean, Europe, South- East Asia, and Western Pacific.

We represented political development using the construct of ‘political democracy’, which is the extent to which a country’s political systems are democratized. As is standard in the human rights index, we measured political democracy using the Polity IV composite index that ranges from − 10 to 10, with higher values suggesting higher degrees of democracy. We dichotomized this variable with countries having a value less than 6 being classified as non-democratic and countries with a value greater or equal to 6 being classified as democratic states. Because we are interested in controlling for the effect of procedural democracy around the time of ratification, we used the Polity IV value that corresponded most closely with each country’s ratification year.

### Statistical analyses

In order to structure the data to account for differences in ratification year across countries, we replaced calendar time scale with a standardized time scale, which we created by ‘zeroing’ the year of ratification. For example, for a country that ratified CEDAW in 1980, 1980 was converted to year-zero and 1985 was converted to year 5 (5-years post-ratification). For a country that ratified CEDAW in 2000, 2000 was converted to year-zero and 2005 was converted to year 5.

We first conducted a set of descriptive statistics, which provided a sense of the sample characteristics, including the time frame of ratification, the geographic distribution of ratifying countries, and country income level.

Next, we characterized the raw trends in life expectancy and mortality rates. We did so first by graphing trends in female life expectancy and mortality rates. Next, we performed paired t-tests to measure differences in life expectancy and mortality rates at the year of CEDAW ratification and 5-years and 10-years after CEDAW ratification.

We also conducted joinpoint regression analysis, which assesses ‘inflection points’; years at which trends in these health outcomes had significantly changed. More specifically, time is modeled as a series of linear trends between meaningful inflection points. The number of inflection points is dictated by trends in the data. Inflection points are fit where the model determines there is a significant change in the linear trend over time since the previous inflection point.

These techniques still fall short of determining the extent to which CEDAW ratification accounts for observed differences in health trends, because while they describe differences in health outcomes over time, they do not assess whether CEDAW is responsible for observed differences. A standard means for isolating the effect of a particular societal condition is through quasi-experimental methods, such as difference-in-differences modeling. These methods compare outcomes in a ‘treatment group (in the present study, this would be the group of countries that have ratified CEDAW) to a ‘control’ group (countries that have not ratified CEDAW, but are otherwise similar to ratifying countries). However, a feature of studying international norms, such as CEDAW ratification, is that precisely because they have been so widely adopted, there is no adequate control group available – no group of (similar) non-ratifying countries - that can be used to approximate an experimental study design.

We therefore used an interrupted-time-series analysis (ITSA), which provides an alternative means for assessing the effect of policies or other societal conditions on health when adequate control groups are not available. ITSA relies on trend data over time. It uses the trend in the health outcome before the policy exposure to mathematically determine what the trend in the health outcome would have been after the policy exposure, if the trend had continued ‘as is’ – meaning, if the policy exposure had not occurred. In other words, it constructs a control group from the treatment group itself. It then compares this mathematically-derived counterfactual trend to the actual observed change in trends after policy exposure occurred. In our analyses, ITSA uses pre-ratification life expectancy and mortality trends to mathematically construct post-ratification life expectancy and mortality trends, had societies not ratified CEDAW, and compares them to the actual change in trends of these outcomes. The difference between the actual and counterfactual trends is the estimated effect of CEDAW on women’s health.

In order to construct these trends, ITSA requires availability of multiple observations of an outcome in both the pre- and post-treatment periods. Due to the availability of health data across a wide range of years, we were able to structure our data set with 5 years of pre-ratification data and up to 20 years of post-ratification on over 80% of countries included (recent ratifiers have less post-ratification data available). We chose 5 years as a pre-ratification window because a longer pre-ratification period might increase the likelihood of contamination by other societal changes.

In order to account for confounding or effect-modification by economic and political development status, we stratified countries based on economic status and presence of democratization. The subgroups for which we ran separate models were: low-income non-democratic countries, low-income democratic countries, middle-income non-democratic countries, middle-income democratic countries, high-income non-democratic countries, and high-income democratic countries. At this early stage of the literature, and with little theoretical or empirical guidance, we remain agnostic regarding the extent of lag impact. For this reason, and reasons of analytic constraints, we did not test lag effects. All analyses were conducted using Stata/SE version 14 (College Station, TX).

## Results

The CEDAW was created in 1979 and ratified between 1980 and 2015 (median ratification year = 1989). 25% of ratifying countries came from the African region, 18% from the Americas, 10% from the Eastern Mediterranean region, 29% from Europe, 6% from South-East Asia, and 12% from the Western Pacific. Just over half of the countries were middle income (*n* = 98) followed by high (*n* = 59) and low-income countries (*n* = 30) (Table 1). Additional file 1 provides more detailed analysis of the distribution of ratification across countries in our sample, and Additional file 2 provides the countries in each stratum. Across income strata, there was no significant difference in mean ratification year, which ranged from 1989 in high-income countries to 1990 in low-income countries. The standard deviation in each income stratum was roughly 8 years. Across strata of democratization, there was a small but significant difference in mean year of ratification (1987 among democratic countries and 1990 among non-democratic countries). Across joint income*democratization strata, there were no significant differences in year of ratification. The earliest mean ratification year (1986, SD: 4.7 years) belonged to democratic, high-income countries, while the latest (1990) belonged to democratic, high-income (1990, SD: 4.7 years) and non-democratic low-income (1990, SD: 8.9 years) and non-democratic middle-income (1990, SD: 7.3 years) countries.

**Table 1:** Characteristics of included countries by UN treaty

	CEDAW
Total Countries (n)	187
Median Ratification Year (range)	1989 (1980–2015)
WHO Region (n, %)	
Africa	47 (25.1)
Americas	34 (18.2)
Eastern Mediterranean	19 (10.2)
Europe	54 (28.9)
South East Asia	11 (5.9)
Western Pacific	22 (11.8)
Country Income Level	
Low	30 (16.0)
Middle	98 (52.4)
High	59 (31.6)
GDP per Capita at Median Ratification Year (mean)	5369.60
GNI per Capita at Median Ratification Year (mean)	5863.31

The mean GDP per capita of countries at the median ratification year for CEDAW was $5863.31. The proportion of democratic countries increased across country income groups with 35% of low-income countries, 51% of middle-income countries, and 78% of high-income countries classified as democratic (Table 1).

**Fig. 1:**
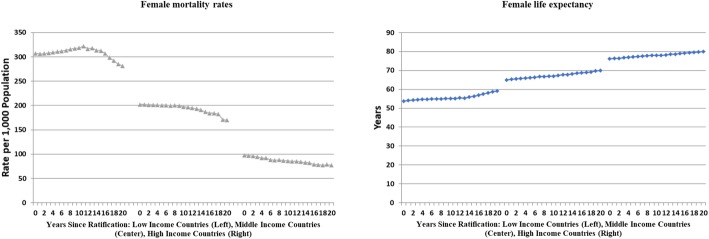
Trends in female mortality and female life expectancy post-CEDAW ratification

Graphing health trends suggested rising life expectancy and declining mortality rates for women across countries in all income groups (Fig. 1). Compared to life expectancy at the year of ratification, all regions and country-income groups had higher average life expectancy at 5 and 10 years post ratification, with the exception of the African region and low-income countries whose life expectancies were not statistically different during this period. Mortality rates suggested a more mixed picture. While they declined in many areas of the world, they were not statistically different in Africa, the Americas, and in low-income or middle-income countries (Table 2).

**Table 2:** Female life expectancy and mortality rates pre- vs. post-CEDAW ratification

	Life Expectancy (years)					Mortality Rate (per 100,000 population)				
	Pre-Ratification	5-Years Post		10-Years Post		Pre-Ratification	5-Years Post		10-Years Post	
WHO Region										
Africa	55.6	55.7	*p* = 0.871	55.2	*p* = 0.886	295.0	317.1	*p* = 0.065	337.5	*p* = 0.034
Americas	68.5	70.2	*p* < 0.001	71.5	p < 0.001	165.1	155.5	*p* = 0.116	151.3	*p* = 0.212
Eastern Mediterranean	69.4	71.3	p < 0.001	71.1	p < 0.001	151.9	137.8	p < 0.001	135.1	p < 0.001
Europe	74.9	76.1	p < 0.001	76.9	p < 0.001	107.9	102.2	*p* = 0.006	96.2	p < 0.001
South East Asia	58.1	61.7	p < 0.001	64.6	p < 0.001	260.0	228.3	*p* = 0.001	202.4	p = 0.001
Western Pacific	67.7	69.7	p < 0.001	71.1	p < 0.001	185.3	163.6	p < 0.001	153.3	p = 0.001
Country Income Level										
Low	53.3	54.5	*p* = 0.068	55.3	*p* = 0.168	308.0	310.0	*p* = 0.874	318.1	*p* = 0.625
Middle	64.7	66.1	p < 0.001	67.0	p < 0.001	202.3	200.0	*p* = 0.653	198.2	*p* = 0.343
High	76.0	77.2	p < 0.001	77.9	p < 0.001	99.7	90.6	p < 0.001	86.3	p < 0.001

The average annual percentage rise in life expectancy ranged from 0.2% among high-income countries (95% CI: 0.20, 0.30) to 0.5% among low-income countries (95%CI: 0.4, 0.5). The average annual percentage decline in mortality rates ranged from − 0.5% in low-income countries (95% CI: -0.6, − 0.3) to − 1.2% in high-income countries (95%CI: -1.5, − 0.9) (Table 3).

**Table 3:** Joinpoint Regression Results Post-CEDAW Ratification

Health Indicator	Mean		Trend 1		Trend 2		Trend 3		Trend 4		AAPC (95% CI)
	Ratification Year	20 Years Post-Ratification	Year	APC (95% CI)	Year	APC (95% CI)	Year	APC (95% CI)	Year	APC (95% CI)	Overall (0–20 Years)
Low Income Countries											
Female Mortality Rate	307.01	281.51	0–3	0.1 (−0.3, 0.6)	3–11	*0.5 (0.4, 0.6)*	11–15	-0.6 (−1.1, −0.2)	15–20	*−2.2 (−2.3, −2.0)*	*−0.5 (−0.6, −0.3)*
Female Life Expectancy	53.78	59.11	0–4	*0.4 (0.3, 0.6)*	4–13	*0.1 (0.1, 0.2)*	13–20	*1.0 (0.9, 1.0)*	–	–	*0.5 (0.4, 0.5)*
Middle Income Countries											
Female Mortality Rate	202.18	169.57	0–10	*−0.2 (−0.2, − 0.1)*	10–18	*−1.2 (− 1.3, − 1.0)*	18–20	*−3.9 (−5.5, − 2.4)*	–	–	*−0.9 (− 1.1, − 0.8)*
Female Life Expectancy	65.00	69.91	0–12	*0.3 (0.3, 0.3)*	12–20	*0.4 (0.4–0.5)*	–	*–*	–	–	*0.4 (0.3, 0.4)*
High Income Countries											
Female Mortality Rate	97.84	77.06	0–7	*−1.6 (− 1.9, − 1.4)*	7–13	*−0.7 (− 1.1, − 0.3)*	13–17	*− 2.0 (− 3.0, − 1.0)*	17–20	− 0.1 (− 1.2, 1.0)	*−1.2 (− 1.5, − 0.9)*
Female Life Expectancy	76.08	79.96	0–7	*0.3 (0.3, 0.3)*	7–12	*0.1 (0.1, 0.2)*	12–15	*0.4 (0.1, 0.7)*	15–20	*0.2 (0.2, 0.3)*	*0.2 (0.2, 0.3)*

*APC* annual % change, *AAPC* average annual % change

Results of joinpoint regression (Table 3) suggested that there were two to four points (depending on the income level of the country) over the 20-year period during which women’s health indicators changed significantly. Among low-income countries, female life expectancy averaged 53.78 years at the year of ratification, and rose to an average of 59.11 years 20 years post ratification. Low-income countries demonstrated three distinct trends in female life expectancy. During the first trend, between the year of ratification and 4 years post ratification, female life expectancy increased by an average of 0.4% (95%CI: 0.3, 0.6). During the second trend, between years 4 and 13 post ratification, the average annual increase in life expectancy slowed to 0.1% (95%CI: 0.1, 0.2). During the final trend, the rate of change in life expectancy increased quite substantially, by 1 % annually on average (95%CI: 0.9, 1.0). Among Middle-income countries, female life expectancy averaged 65 years at the year of ratification, and grew to 69.91 years 20 years post ratification. Among these countries, there were two distinct trends, which had an annual average increase in life expectancy of 0.3 to 0.4%. Among high-income countries, female life expectancy averaged 76.08 years at the year of ratification, and grew to 79.96 years 20 years later. During that time, four distinct trends were detected. The first was a trend for the first 7 years post ratification, during which female life expectancy increased annually by an average of 0.3% (95%CI: 0.3, 0.3). The next trend occurred from year 7 to year 12, during which life expectancy increased at a slightly slower pace, by an average annual rate of 0.1% (95%CI: 0.1, 0.2). From year 12 to 15 post-ratification, the rate of increase life expectancy in low-income countries increase to an annual average of 0.4% (0.1, 0.7). Finally, from years 15 to 20, the average annual increase slowed to an average of 0.2% (95%CI: 0.2, 0.3).

Female mortality rates exhibited between three and five distinct trends, depending on the income level of the country (Table 3). In low-income countries, female mortality rates averaged 307.01 deaths per 100,000 population, and decreased to 281.51 deaths per 100,000 population 20 years post ratification. During the initial 11 years post ratification mortality rates increased (by an average of 0.1% during the first 3 years post ratification (95%CI: -0.3, 0.6) and then by an average of 0.5% for the next 8 years (95%CI: 0.4, 0.6). During the last 4 years (from year 11 to 15 post ratification), mortality rates declined by an annual average of 0.6% (95%CI: -1.1, − 0.2). Between years 15 and 20, they declined even more rapidly, by an annual average rate of 2.2% (95%CI: -2.3, − 2.0). Among middle-income countries, mortality rates averaged 202.18 deaths per 100,000 population, and declined, to 169.57 deaths per 100,000 population, 20 years after ratification. Three distinct trends emerged (from ratification year 10 post ratification, from years 10 to 18 post ratification, and from year 18 to year 20). Each trend accelerated the rate of decline in mortality rates, from an initial average annual decline of 0.2% per year (95% CI: -0.2,-0.2) to a decline of 3.9% per year (95% CI: -5.5, − 2.4). Among high income countries, female mortality rates averaged 97.84 deaths per 100,000 population at the year of ratification, and shrunk to 77.06 deaths per 100,000 20 years post ratification. Each of the four observed trends demonstrated declines, ranging from 0.7% (from year 7 to 13) to 2 % (from year 13 to year 17).

**Fig. 2:**
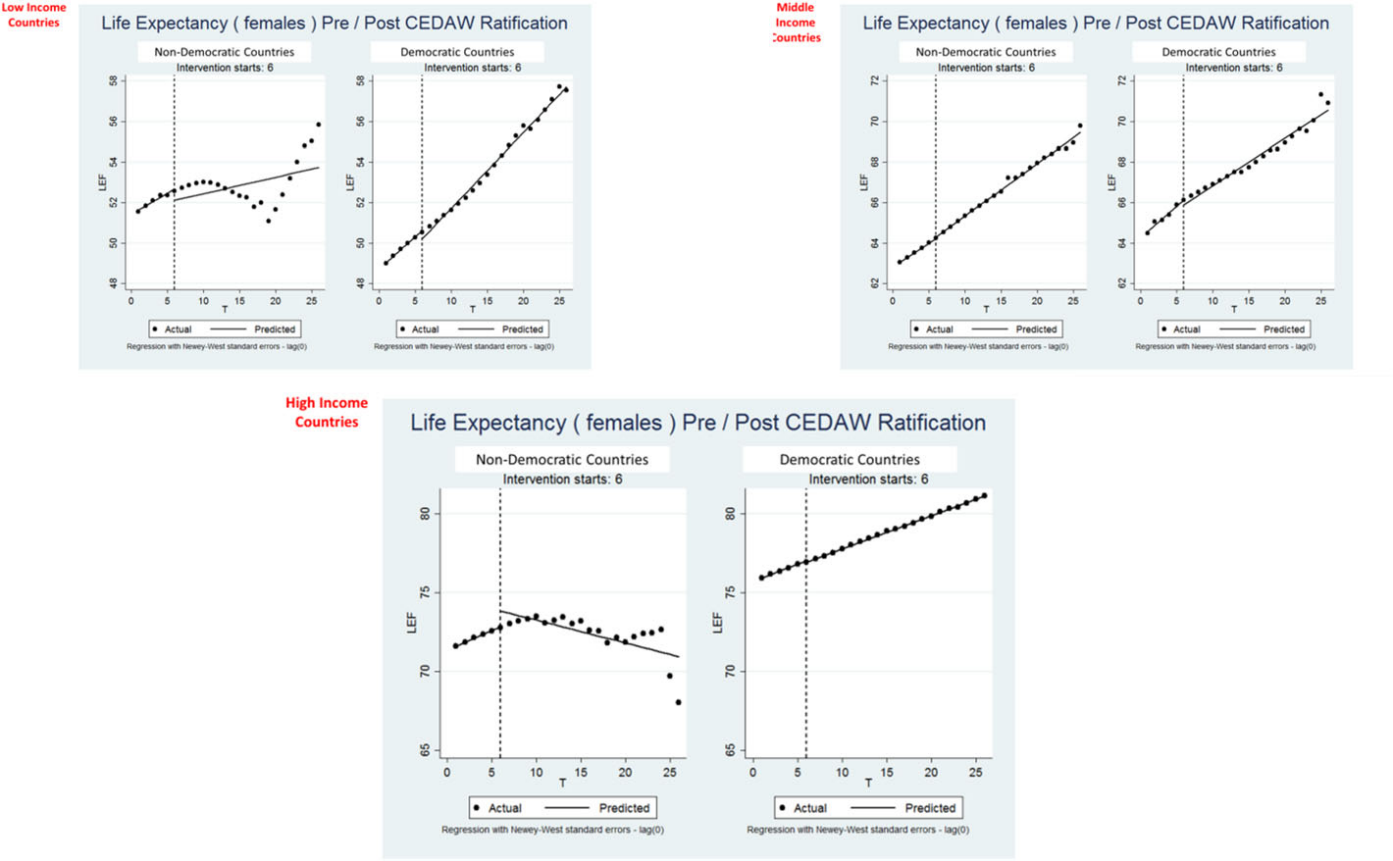
Interrupted time series trends for female life expectancy, pre- vs. post-CEDAW ratification

**Fig. 3:**
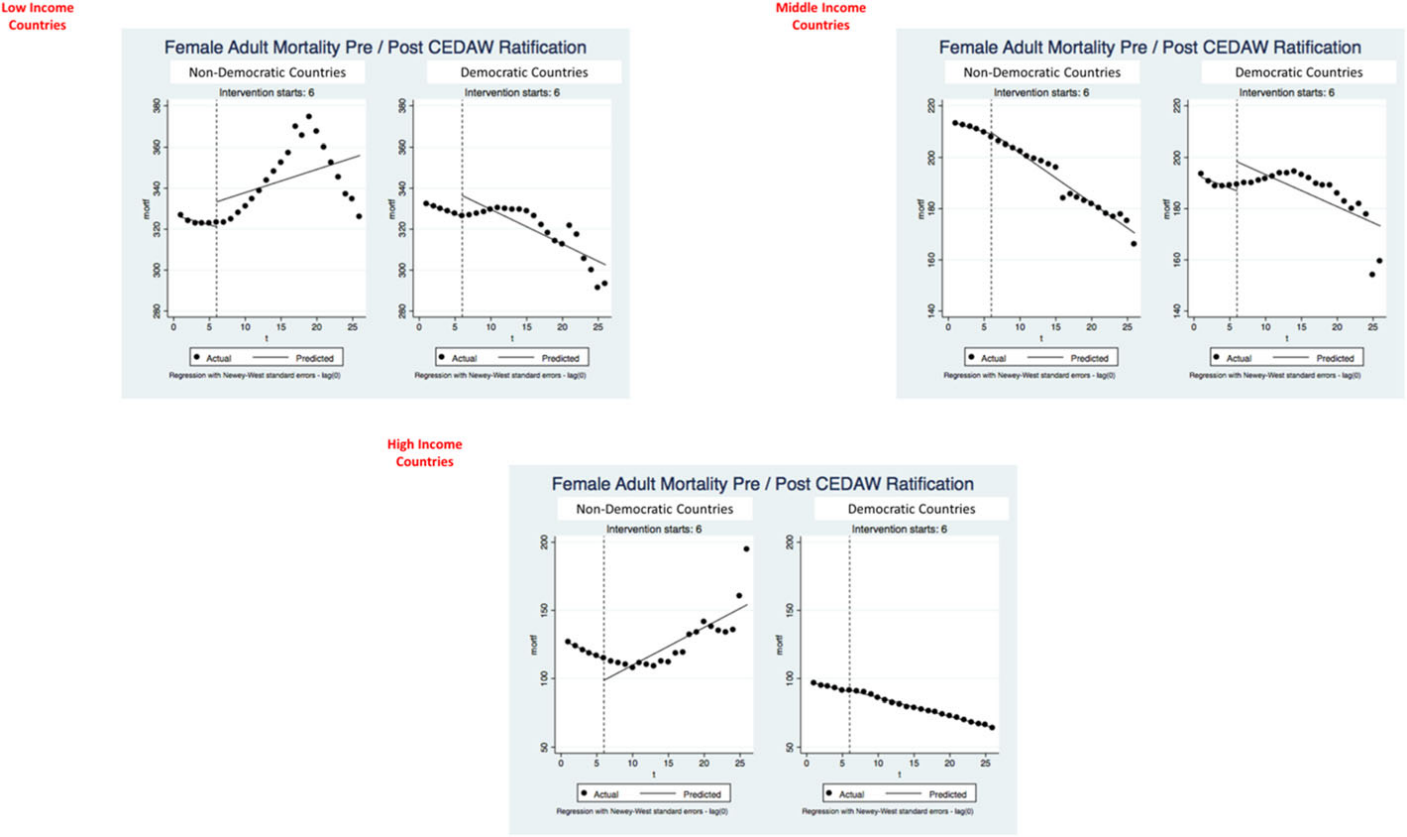
Interrupted time series trends for female mortality rates, pre- vs. post-CEDAW ratification

Results of the interrupted times series analyses presented quite a mixed picture (Figs. 2 and 3 and Table 4). In the stratum of low-income countries, CEDAW was not associated with improvements in female life expectancy for non-democratic countries. However, CEDAW ratification (and a sustained effect of ratification over time) was evident among the democratic countries. CEDAW ratification appeared to have no impact on female life expectancy among the middle-income countries. Among the high-income countries, the impact of CEDAW was largely significant and positive. CEDAW had much the same association with female mortality rates, with the exception of middle-income, non-democratic countries, for which CEDAW was associated with significant declines in mortality rates.

**Table 4:** Summary of Interrupted Time Series Analysis

	Low Income Countries		Middle Income Countries		High Income Countries	
	Non-Democratic	Democratic	Non-Democratic	Democratic	Non-Democratic	Democratic
Female Life Expectancy						
Significant Ratification Effect	No	YES	No	No	YES	YES
Significant Ratification Effect Over Time	No	YES	No	No	YES	No
Female Mortality Rate						
Significant Ratification Effect	No	YES	YES	No	YES	YES
Significant Ratification Effect Over Time	No	YES	YES	No	YES	YES

## Discussion

Our analyses suggests that, while population indicators of women’s health have improved since CEDAW ratification [16], the impact of CEDAW ratification itself was not uniform. In the low-income stratum, CEDAW was effective in democratic, but not in non-democratic countries. In the middle-income stratum, CEDAW was largely ineffective. In the high-income stratum, CEDAW was largely effective.

Our findings are somewhat perplexing. The intention of CEDAW is to improve the status of women, which public health theory predicts should improve population indicators of women’s health [1]. And yet, CEDAW ratification was not uniformly associated with better life expectancy or mortality rates. With few previous studies on this topic, we can only offer general speculations to explain our findings.

The first possibility is that CEDAW ratification does not necessarily translate into improved social and economic conditions for women. In other words, there may be a disconnect between ratification and the ‘real’ implementation of policies and programs that have the potential to foster the elimination (or even reduction) in discrimination against women. Our binary indicator of ratification did not assess the post-ratification actions (or inactions) of countries.

The second possibility is that the effects of CEDAW are too weak, independent of other societal conditions, to register an effect on population-levels of women’s health. This is suggested, for example, by the fact that CEDAW appears to be associated with improvements with women’s health in the high-income strata, where other societal conditions provide essential preconditions. It is also suggested by the finding that CEDAW was not effective in low-income countries that are not democratized, but was effective in those which are, suggesting democratization may also be a highly influential precondition. On the other hand, it is difficult to make sense of the lack of effect across the middle-income stratum.

The third possibility is that confounders for which we were unable to account are complicating our ability to assess the ‘true’ effect of CEDAW. There are several sources of confounding that are of concern. Over the course of time that CEDAW was ratified, many other global changes took place. Perhaps most notably, globalization of markets and trade ramped up considerably. Specific secular changes had also occurred, which may or may not be account for by globalization [20]: decline in poverty rates, rise in income inequality, improvements in medical care. Because these factors may influence women’s health, and are possibly associated with CEDAW ratification, our study might be biased by not including them. As aforementioned, we are not entirely sure whether these should be considered confounders or effect modifiers. Moreover, for mortality rates, we were unable to locate age-adjusted rates, and therefore changes in the age distribution over time were also unaccounted for, and may have biased our results. Of note, because our results stood up over a range of ratification years, any potential societal-level confounders are likely to be ones that unfolded over time, rather than sudden societal shocks.

The time-varying nature of national income and, potentially, of democratization, also may have posted a problem. The implication is that countries may have moved in and out of strata over the period of analysis, which may mean that, by using the year of ratification as the year by which to categorize countries by income and democratization, some misclassification may have occurred. We believe, however, this was minimal.

Indeed, the main limitation of our study is our limited means for addressing threats to validity, namely those introduced by sources of unmeasured confounding. This was principally attributable to the fact that we were unable to incorporate a ‘true’ control group, which would tell us what happened to women’s life expectancy and mortality trends in similar countries that did not undergo CEDAW ratification [6]. However, as discussed earlier, this was simply not possible, given that state commitment to CEDAW is an international norm – it has been broadly adopted - and thus there is a true lack of available control countries [19]. This paper thus raises interesting methodological questions regarding how to test the effects of norms.

## Conclusions

Our findings yielded mixed effects for CEDAW, indicating either that CEDAW’s impact on women’s health is highly dependent on other societal conditions, such as income level, democratization, or a host of other variables, for which we did not account or, that CEDAW does not have an especially large independent impact on women’s health. Our study provides a way to assess the effects of norms, and also how to examine if the effects of norms, even ostensibly widely-held ones, vary in the ways in which they manifest in different countries. Future studies should continue to investigate the associations between international human rights treaties and population health outcomes.

## Additional files

Additional file 1: **Table S1.** Mean ratification year across groups in stratified analyses. (DOCX 14 kb)

Additional file 2: **Table S2.** Distribution of ratification year across stratified groups. (DOCX 22 kb)

## Abbreviations

CEDAW: Convention on the Elimination of All Forms of Discrimination Against Women
GDP: Gross domestic product
GNI: Gross national income
ITSA: Interrupted time series analysis
