# Supplementary material for: Can the health effects of widely-held societal norms be evaluated? An analysis of the United Nations convention on the elimination of all forms of discrimination against women (UN-CEDAW)
Source: BMC Public Health. 2019 Mar 8;19:279. doi: 10.1186/s12889-019-6607-6 (PMC6408842; doi:10.1186/s12889-019-6607-6)
Supplement: Supplementary file 1 — Table S1. Mean ratification year across groups in stratified analyses. (DOCX 14 kb) [file 12889_2019_6607_MOESM1_ESM.docx]

**Table S1. Mean ratification year across groups in stratified analyses**

|  | Mean ratification year (SD) |  | *p-value* |
| --- | --- | --- | --- |
|  |  |  |  |
| **Income** |  |  | 0.471 |
| *Low* | 1990 (7.6) |  |  |
| *Middle* | 1989 (8.0) |  |  |
| *High* | 1989 (7.7) |  |  |
|  |  |  |  |
| **Democratic Status** |  |  | 0.0048 |
| *Democratic* | 1987 (6.2) |  |  |
| *Non-democratic* | 1990 (8.3) |  |  |
|  |  |  |  |
| ***Income-Democratic Status*** |  |  |  |
| Democratic |  |  | 0.113 |
| *Low* | 1990 (4.7) |  |  |
| *Middle* | 1988 (7.3) |  |  |
| *High* | 1986 (4.7) |  |  |
| Non-democratic |  |  | 0.349 |
| *Low* | 1990 (8.9) |  |  |
| *Middle* | 1990 (7.3) |  |  |
| *High* | 1996 (10.2) |  |  |
