# Supplementary material for: Can the health effects of widely-held societal norms be evaluated? An analysis of the United Nations convention on the elimination of all forms of discrimination against women (UN-CEDAW)
Source: BMC Public Health. 2019 Mar 8;19:279. doi: 10.1186/s12889-019-6607-6 (PMC6408842; doi:10.1186/s12889-019-6607-6)
Supplement: Supplementary file 2 — Table S2. Distribution of ratification year across stratified groups. (DOCX 22 kb) [file 12889_2019_6607_MOESM2_ESM.docx]

**Table S2. Distribution of ratification year across stratified groups**

| **Democratic** | | | | | |  | **Non-democratic** | | | | | |
| --- | --- | --- | --- | --- | --- | --- | --- | --- | --- | --- | --- | --- |
|  |  |  |  |  |  |  |  |  |  |  |  |  |
| **Low** |  | **Middle** |  | **High** |  |  | **Low** |  | **Middle** |  | **High** |  |
| Liberia | 1984 | Cabo Verde | 1980 | Hungary | 1980 |  | Ethiopia | 1981 | China | 1980 | Russian Federation | 1981 |
| Guinea-Bissau | 1985 | Guyana | 1980 | Poland | 1980 |  | Haiti | 1981 | Cuba | 1980 | Venezuela, RB | 1983 |
| Malawi | 1987 | El Salvador | 1981 | Portugal | 1980 |  | Rwanda | 1981 | Belarus | 1981 | Equatorial Guinea | 1984 |
| Sierra Leone | 1988 | Mexico | 1981 | Sweden | 1980 |  | Guinea | 1982 | Bhutan | 1981 | Kuwait | 1994 |
| Madagascar | 1989 | Mongolia | 1981 | Canada | 1981 |  | Togo | 1983 | Ecuador | 1981 | Singapore | 1995 |
| Nepal | 1991 | Nicaragua | 1981 | Norway | 1981 |  | Mali | 1985 | Egypt, Arab Rep. | 1981 | Saudi Arabia | 2000 |
| Benin | 1992 | Panama | 1981 | Uruguay | 1981 |  | Tanzania | 1985 | Lao PDR | 1981 | Bahrain | 2002 |
| Burundi | 1992 | Philippines | 1981 | Austria | 1982 |  | Uganda | 1985 | Sri Lanka | 1981 | United Arab Emirates | 2004 |
| Comoros | 1994 | Bulgaria | 1982 | Australia | 1983 |  | Congo, Dem. Rep. | 1986 | Ukraine | 1981 | Oman | 2006 |
| Mozambique | 1997 | Colombia | 1982 | Denmark | 1983 |  | Burkina Faso | 1987 | Congo, Rep. | 1982 | Qatar | 2009 |
| Niger | 1999 | Dominican Republic | 1982 | France | 1983 |  | Central African Republic | 1991 | Vietnam | 1982 |  |  |
|  |  | Guatemala | 1982 | Greece | 1983 |  | Zimbabwe | 1991 | Gabon | 1983 |  |  |
|  |  | Peru | 1982 | Korea, Rep. | 1984 |  | Cambodia | 1992 | Bangladesh | 1984 |  |  |
|  |  | Romania | 1982 | Spain | 1984 |  | Gambia, The | 1993 | Yemen, Rep. | 1984 |  |  |
|  |  | Honduras | 1983 | Argentina | 1985 |  | Chad | 1995 | Nigeria | 1985 |  |  |
|  |  | Brazil | 1984 | Belgium | 1985 |  | Eritrea | 1995 | Thailand | 1985 |  |  |
|  |  | Indonesia | 1984 | Cyprus | 1985 |  | Korea, Dem. Rep. | 2001 | Angola | 1986 |  |  |
|  |  | Jamaica | 1984 | Germany | 1985 |  | Afghanistan | 2003 | Iraq | 1986 |  |  |
|  |  | Kenya | 1984 | Ireland | 1985 |  | South Sudan | 2015 | Libya | 1989 |  |  |
|  |  | Mauritius | 1984 | Italy | 1985 |  |  |  | Jordan | 1992 |  |  |
|  |  | Senegal | 1985 | Japan | 1985 |  |  |  | Armenia | 1993 |  |  |
|  |  | Tunisia | 1985 | New Zealand | 1985 |  |  |  | Bosnia and Herzegovina | 1993 |  |  |
|  |  | Turkey | 1985 | Finland | 1986 |  |  |  | Morocco | 1993 |  |  |
|  |  | Zambia | 1985 | United Kingdom | 1986 |  |  |  | Suriname | 1993 |  |  |
|  |  | Costa Rica | 1986 | Chile | 1989 |  |  |  | Tajikistan | 1993 |  |  |
|  |  | Ghana | 1986 | Trinidad and Tobago | 1990 |  |  |  | Cameroon | 1994 |  |  |
|  |  | Paraguay | 1987 | Estonia | 1991 |  |  |  | Azerbaijan | 1995 |  |  |
|  |  | Bolivia | 1990 | Israel | 1991 |  |  |  | Cote d'Ivoire | 1995 |  |  |
|  |  | Namibia | 1992 | Netherlands | 1991 |  |  |  | Fiji | 1995 |  |  |
|  |  | India | 1993 | Croatia | 1992 |  |  |  | Malaysia | 1995 |  |  |
|  |  | Albania | 1994 | Latvia | 1992 |  |  |  | Papua New Guinea | 1995 |  |  |
|  |  | Georgia | 1994 | Slovenia | 1992 |  |  |  | Uzbekistan | 1995 |  |  |
|  |  | Macedonia, FYR | 1994 | Czech Republic | 1993 |  |  |  | Algeria | 1996 |  |  |
|  |  | Moldova | 1994 | Slovak Republic | 1993 |  |  |  | Myanmar | 1997 |  |  |
|  |  | Lesotho | 1995 | Lithuania | 1994 |  |  |  | Turkmenistan | 1997 |  |  |
|  |  | South Africa | 1995 | Switzerland | 1997 |  |  |  | Djibouti | 1998 |  |  |
|  |  | Botswana | 1996 |  |  |  |  |  | Kazakhstan | 1998 |  |  |
|  |  | Pakistan | 1996 |  |  |  |  |  | Mauritania | 2001 |  |  |
|  |  | Kyrgyz Republic | 1997 |  |  |  |  |  | Syrian Arab Republic | 2003 |  |  |
|  |  | Lebanon | 1997 |  |  |  |  |  | Swaziland | 2004 |  |  |
|  |  | Serbia | 2001 |  |  |  |  |  |  |  |  |  |
|  |  | Solomon Islands | 2002 |  |  |  |  |  |  |  |  |  |
|  |  | Timor-Leste | 2003 |  |  |  |  |  |  |  |  |  |
|  |  | Montenegro | 2006 |  |  |  |  |  |  |  |  |  |
